# Challenges and perspectives in preventing and treating obesity

**DOI:** 10.1590/1516-3180.2024.1426.13062024

**Published:** 2024-10-11

**Authors:** Denis Pajecki, Paulo Manuel Pêgo Fernandes

**Affiliations:** I Department of Gastroenterology, Hospital das Clínicas, Faculdade de Medicina, Universidade de São Paulo, São Paulo, Brazil.; II Vice-Director, School of Medicine, University of São Paulo, São Paulo, Brazil; Full Professor, Department of Cardiopulmonary Diseases, Faculdade de Medicina, Universidade de São Paulo, São Paulo, Brazil; Director of the Scientific Department, Associação Paulista de Medicina, São Paulo, Brazil.

The American Medical Association recognized obesity as a chronic disease in 2013, followed by the World Health Organization. At that time, the global incidence and prevalence of obesity had already been progressively increasing for at least three decades. This scenario has not changed; recent data estimate that 1 billion people worldwide will be obese by 2030. The Brazilian Institute of Geography and Statistics has reported that 25% of the adult Brazilian population lives with the disease, a percentage that has quadrupled in children and adolescents since the 1990s.^1^

The etiology of obesity is mostly related to a combination of genetic and environmental factors, such as diet and lifestyle that influence people at different levels, resulting in heterogeneous presentations. Genome-wide association studies have identified hundreds of single nucleotide polymorphisms associated with body mass index (BMI), fat distribution, body composition, and other phenotypic differences. The pathophysiology of obesity involves dysfunctional energy balance, which is regulated by several complex metabolic pathways that interfere with appetite control and energy expenditure, leading to body fat accumulation. Metabolic adaptation phenomena such as increased orexigenic hormone secretion and decreased energy expenditure justify a tendency to regain weight when treatment is interrupted.^2^

However, both the general population and health professionals continue to view the disease as a behavioral disorder. This bias, or stigma, affects the search for and prescription of treatment blurs the distinction between prevention and treatment, and hinders investment in public policies aimed at disease control. Consequently, the incidence of severe obesity—defined by a higher BMI or the presence of comorbidities—rises exponentially, resulting in higher mortality rates from cardiovascular disease and cancer in affected people, deterioration in quality of life, and greater healthcare costs. Recent data indicate that the Brazilian Unified Health System has annual direct costs of BRL 1.5 billion and indirect costs, related to treating associated comorbidities, of up to BRL 190 billion. In the supplementary health sector, direct and indirect spending on obesity will account for an estimated 46% of claims by 2030.^3^

Improving eating habits and lifestyle forms the basis of *prevention* and public policies to control obesity. Recent taxation of sugary beverages under the Selective Tax (PEC 45/2019) represents significant progress; however, excluding ultra-processed foods from the proposal was a missed opportunity. For decades, the United States has attempted to change the habits of children and adolescents through tax policies and projects to reduce the incidence of obesity, without success.^4^

However, the belief that diet and physical activity are the most effective *treatment* measures still prevails. Although these measures are necessary and may be sufficient for a few patients, especially those with milder disease, they have poor mean long-term effectiveness in patients with established and more severe disease. More than a weight loss method, patients with obesity require long-term strategies to maintain treatment results.^2^

Thus, medications acting on different aspects of appetite may be necessary. In the past, obesity was treated with nonselective anorectic medications that had many adverse effects, reinforcing the stigma associated with the disease and its treatment. Modern medications are more selective and have a good safety profile, opening new perspectives for long-term disease control. These medications include glucagon-like peptide-1 agonists and their combination with other gastrointestinal hormone agonists, which have shown mean weight losses of more than 15% in controlled studies, delivering unprecedented results. Evidence that effective obesity treatment with these medications leads to fewer cardiovascular events and better control of other comorbidities has encouraged physicians from several specialties to prescribe this type of treatment to their patients. Facilitating access to these medications remains challenging due to their high cost; encouraging patients to engage in prolonged treatment associated with lifestyle changes is as challenging.^5^

Treatment with bariatric and metabolic surgery is reserved for severe forms of obesity. Historically, the concept of severe obesity was closely related to a patient’s BMI, with a basis for surgical indication criteria established more than three decades ago (i.e., BMI above 40 kg/m^2^ or above 35 kg/m^2^ with comorbidities). More recently, severity scoring systems such as the Edmonton Obesity Scoring System have shown that the presence and severity of comorbidities are more sensitive factors for predicting cardiovascular risk and mortality. In this context, surgery may be indicated for patients with a BMI of between 30 and 35 kg/m^2^ with poorly controlled comorbidities.^2^

Over the last 30 years, the field of surgical treatment has progressed considerably with the advent of minimally invasive access (i.e., laparoscopic surgery) and improved treatment safety. Surgical morbidity and mortality have significantly reduced and are now comparable to common surgical procedures such as hysterectomy, hernia correction, or orthopedic procedures. Controlled and observational studies involving thousands of patients have demonstrated the long-term effectiveness of the treatment, with 1-year weight loss of 30% to 40% and long-term weight loss of 20% to 30% reported, depending on the technique used. These studies have reported the control of comorbidities such as type 2 diabetes and high blood pressure, with operated patients presenting an increased life expectancy of 6 years.^2^

However, patient referrals for surgery by physicians from other disciplines remain low, with most patients seeking treatment on their own and often struggling with difficult access, especially in the Brazilian Unified Health System.^3^ The stigma surrounding surgery is high, with this treatment often viewed as futile or as representing patient failure.^4^ Therapeutic options for controlling obesity have progressed in several fields. However, understanding that treatments do not compete, but rather that their incorporation into a treatment strategy depends on the individual condition of each patient, is vital. Surgical treatment associated with multidisciplinary care is a safe and effective option for more severely obese patients and should be used when other alternatives fail.^5^

## References

[1] GBD 2021 Diseases and Injuries Collaborators (2024). Global incidence, prevalence, years lived with disability (YLDs), disability-adjusted life-years (DALYs), and healthy life expectancy (HALE) for 371 diseases and injuries in 204 countries and territories and 811 subnational locations, 1990-2021: a systematic analysis for the Global Burden of Disease Study 2021. Lancet.

[2] Syn NL, Cummings DE, Wang LZ (2021). Association of metabolic-bariatric surgery with long-term survival in adults with and without diabetes: a one-stage meta-analysis of matched cohort and prospective controlled studies with 174 772 participants. Lancet.

[3] Instituto de Estudos de Saúde Suplementar (2022). Como o aumento da prevalência da obesidade entre beneficiários pode impactar a sustentabilidade da saúde suplementar.

[4] Sharma AM, Kushner RF (2009). A proposed clinical staging system for obesity. Int J Obes.

[5] Eisenberg D, Shikora SA, Aarts E (2023). 2022 American Society of Metabolic and Bariatric Surgery (ASMBS) and International Federation for the Surgery of Obesity and Metabolic Disorders (IFSO) Indications for Metabolic and Bariatric Surgery. Obes Surg.

